# Complete Genome Sequence of *Pseudomonas aeruginosa* FA-HZ1, an Efficient Dibenzofuran-Degrading Bacterium

**DOI:** 10.1128/genomeA.01634-16

**Published:** 2017-02-16

**Authors:** Fawad Ali, Haiyang Hu, Ping Xu, Hongzhi Tang

**Affiliations:** State Key Laboratory of Microbial Metabolism and School of Life Sciences and Biotechnology, Shanghai Jiao Tong University, Shanghai, People’s Republic of China

## Abstract

*Pseudomonas* sp. FA-HZ1, an efficient dibenzofuran-degrading bacterium, was isolated from landfill leachate. Here, we present the complete genome sequence of strain FA-HZ1, which contains only one circular chromosome. The complete genome sequence will be essential for revealing the molecular mechanisms of dibenzofuran degradation.

## GENOME ANNOUNCEMENT

*Pseudomonas* sp. FA-HZ1, isolated from landfill leachate, has the ability to degrade dibenzofuran (DBF). It can utilize DBF as a sole carbon source to grow. To date, no genome of a *Pseudomonas* species with the efficient ability to degrade DBF has been completely sequenced. In order to explain the mechanism of DBF degradation, we present the complete genome sequence of strain FA-HZ1. The complete genome sequence will be essential for the molecular mechanism of DBF degradation. It will provide useful genetic information to other microorganisms that are involved in DBF degradation.

The complete genome was sequenced with the Pacific Biosciences (PacBio) RS II sequencer (Pacific Biosciences, USA). The generated sequencing reads were *de novo* assembled using the PacBio RS hierarchical genome assembly process (HGAP) version 2 in SMRT analysis version 2.3 (https://github.com/PacificBiosciences/SMRT-Analysis).

The 6,838,112-bp genome is composed of one circular chromosome with a G+C content of 66%. The coding region is about 6,389 bp in length (0.093% of the genome), in which 6,064 protein-coding sequences were identified. The genome also encodes 12 rRNAs, 66 tRNAs, and 4 ncRNAs, all of which make up 0.001% of the genome. Through rapid genome annotation using the RAST server, 158 genes that are related to the metabolism of aromatic compounds were found. Important genes, such as the 2,3-dihydroxybipheyle-1,2-dioxygenase gene *bphC* that is involved in the complete degradation of DBF (1) were annotated in the genome sequence. The genome also contains other enzymes such as ferredoxin reductase, which functions as an electron transporter in DBF degradation (2), as well as monooxygenase and dehydrogenase. The genes associated with benzoate degradation, along with some membrane-transport proteins and transcriptional regulators, were also annotated in the genome of strain FA-HZ1. Moreover, 110 genes associated with motility and chemotaxis were found, which may help the bacterium to target and degrade the chemical compounds (3).

### Accession number(s).

The genome sequence of *P. aeruginosa* sp. FA-HZ1 has been deposited in GenBank under the accession number CP017353.

## References

[1] Wesche J, Hammer E, Becher D, Burchhardt G, Schauer F 2005 The *bphC* gene-encoded 2,3-dihydroxybiphenyl-1,2-dioxygenase is involved in complete degradation of dibenzofuran by the biphenyl-degrading bacterium *Ralstonia* sp. SBUG 290. J Appl Microbiol 98:635–645. doi:10.1111/j.1365-2672.2004.02489.x. 15715866

[2] Edith C, Clemence R, David J, Jan M 2012 Genome-wide analysis of salicylate and dibenzofuran metabolism in *Sphingomonas* *wittichii* RW1. FEMS Microbiol 3:300. doi:10.3389/fmicb.2012.00300. PMC3425912 22936930

[3] Sampedro I, Parales RE, Krell T, Hill JE 2015 *Pseudomonas* chemotaxis. FEMS Microbiol Rev 39:17–46. doi:10.1111/1574-6976.12081. 25100612

